# Effect of Iron Tailing Powder-Based Ternary Admixture on Acid Corrosion Resistance of Concrete

**DOI:** 10.3390/ma16103688

**Published:** 2023-05-12

**Authors:** Bing Zhang, Yannian Zhang, Wenliang Liu, Xiaowei Gu, Qingjie Wang, Shaowu Zhang, Jian Gao

**Affiliations:** 1 School of Civil Engineering, Jilin University of Architecture and Technology, Changchun 130114, China; 2 School of Civil Engineering, Shenyang Jianzhu University, Shenyang 110168, China; 3 Science and Technology Innovation Center of Smart Water and Resource Environment, Northeastern University, Shenyang 110819, China; 4 China Railway Construction Bridge Engineering Bureau Group 4th Engineering Co., Ltd., Harbin 150008, China

**Keywords:** iron tailing powder, fly ash, lithium slag, acid erosion, compressive strength

## Abstract

Exposure of concrete to acidic environments can cause the degradation of concrete elements and seriously affect the durability of concrete. As solid wastes are produced during industrial activity, ITP (iron tailing powder), FA (fly ash), and LS (lithium slag) can be used as admixtures to produce concrete and improve its workability. This paper focuses on the preparation of concrete using a ternary mineral admixture system consisting of ITP, FA, and LS to investigate the acid erosion resistance of concrete in acetic acid solution at different cement replacement rates and different water–binder ratios. The tests were performed by compressive strength analysis, mass analysis, apparent deterioration analysis, and microstructure analysis by mercury intrusion porosimetry and scanning electron microscopy. The results show that when the water–binder ratio is certain and the cement replacement rate is greater than 16%; especially at 20%, the concrete shows strong resistance to acid erosion; when the cement replacement rate is certain and the water–binder ratio is less than 0.47; especially at 0.42, the concrete shows strong resistance to acid erosion. Microstructural analysis shows that the ternary mineral admixture system composed of ITP, FA, and LS promotes the formation of hydration products such as C-S-H and AFt, improves the compactness and compressive strength of concrete, and reduces the connected porosity of concrete, which can obtain good overall performance. In general, concrete prepared with a ternary mineral admixture system consisting of ITP, FA, and LS has better acid erosion resistance than ordinary concrete. The use of different kinds of solid waste powder to replace cement can effectively reduce carbon emissions and protect the environment.

## 1. Introduction

Concrete is one of the most extensively used building materials in the world, and its durability is exceptionally significant in structural design, especially in infrastructure design, where the performance of concrete exposed to acidic environments is one of the critical issues of concrete durability [1,2]. Exposure of concrete to acidic environment will reduce the performance of infrastructure, shorten the service life and increase the maintenance cost.

Regarding the improvement of the operational performance of concrete structures in acidic environments, some studies have shown that using different kinds of admixtures and new materials can improve the performance of concrete. In addition, the use of biocide to inhibit the growth of acid bacteria is also one of the effective ways to achieve this [3,4]. The effects of cement type, water–cement ratio (W/C) [5,6], polymer materials, aggregate type, and supplementary cementitious materials (SCMs) [7,8,9] of acid erosion resistance of concrete have been investigated in some previous studies. Alexander et al. [10] studied ordinary Portland cement concrete and calcium aluminate cement concrete under the conditions of biological acids in sewers and showed that calcium aluminate cement concrete is significantly better than ordinary Portland cement concrete in terms of acid erosion resistance. Kim et al. [11] tested cement mortar samples with a W/C of 0.45 as a control case and evaluated the durability performance at W/C of 0.45 to 0.60. The findings showed that the durability performance of cement mortar decreased significantly with the increase in porosity with the rise in W/C. Wang et al. [12] researched concrete incorporated with super absorbent polymers (SAP) under the effect of acid rain erosion. The studies showed that with the addition of SAP, the erosive effect of acid rain on concrete diminished. The added SAP promoted hydration, reduced deterioration due to pore deformation, improved the internal pore structure of concrete, and enhanced the acid erosion resistance of concrete. Araghi et al. [13] investigated sulfuric acid erosion on concrete using polyethene terephthalate (PET) particles as replacement aggregates and determined that developing the number of PET particles as replacement aggregates in concrete resulted in less crushing load and loss of weight. Ultrasonic pulse velocity values and concrete containing PET particles had better resistance to sulfuric acid erosion performance. Roy et al. [9] examined the effects of mortars prepared using silica fume, fly ash, and metakaolin, respectively, as SCMs in different acid corrosion environments and showed that the addition of silica fume, fly ash, and metakaolin, respectively, improved the acid erosion resistance of concrete. Nowadays, the application of SCMs [14,15,16,17] is effectively practiced, and the commonly used SCMs are mainly industrial solid wastes [18,19,20,21]. A series of research results have shown that the use of industrial solid wastes as SCMs can improve the performance of concrete, which is of great significance to save natural energy and protect the environment. Bakharev et al. [22] explored the durability of alkali-activated slag concrete under acid erosion. Analysis of the evolution of compressive strength, degradation products, and microstructural changes in concrete shows that activated slag concrete has high acid resistance in acidic environments and outperforms the durability of ordinary concrete of the same grade. Cheng et al. [23] prepared concrete by replacing cement with iron tailings powder as an admixture. The permeability, frost resistance, and carbonation resistance of the concrete were tested, which shown that concrete made by substituting part of the cement with mechanochemically activated iron tailings has better durability than ordinary concrete. Zhai et al. [24] added a certain amount of lithium slag powder as a kind of SCM in the cement system, and disciovered that the combination of appropriate amount of lithium slag powder could make the slurry structure denser, enhance the permeability resistance of concrete, and further improve the durability of concrete. Goyal et al. [25] performed an experimental study using a binary mixture of silica fume and fly ash versus silica fume only as a kind of SCM to monitor the corrosion process by measuring mass loss and compressive strength in an aggressive chemical environment. The study reported that the binary mixture of silica fume and fly ash demonstrated superior erosion resistance to the mixture containing only silica fume as a kind of SCM.

Although the use of SCMs to enhance the durability of concrete has been extensively studied [26,27], most studies have focused on a single mineral admixture, while the nature of the mineral admixture can also negatively affect the concrete, such as the low volcanic ash activity of some mineral admixtures [28,29,30], which is not conducive to the improvement of concrete strength. In addition, compared with using a single mineral admixture, there is significantly less research on the durability of concrete with multiple mineral admixtures. Therefore, in this study, concrete was produced using a ternary mineral admixture system consisting of industrial solid waste ITP, FA, and LS, immersed in a low pH acetic acid environment for durability studies. On this basis, the effects of different cement replacement rates (CRRs) and different water–binder ratios (w/b) on the compressive strength, compressive strength loss, mass loss, and apparent deterioration of concrete were analyzed, while the pore space and hydration products of ternary mineral admixture concrete were investigated using mercury intrusion porosimetry (MIP) and scanning electron microscopy (SEM) techniques to support the conclusions obtained from the experiments.

## 2. Materials and Methods

### 2.1. Materials

**Figure 1:**
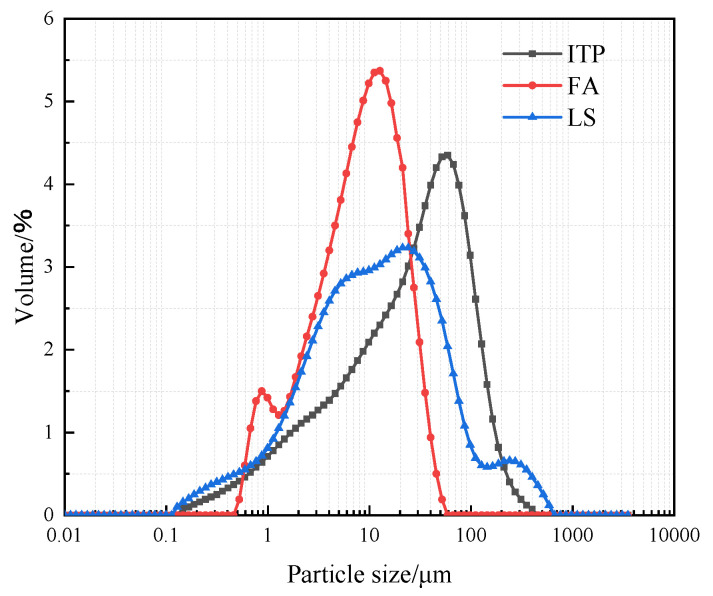
The particle size distribution of ITP, FA, and LS.

**Figure 2:**
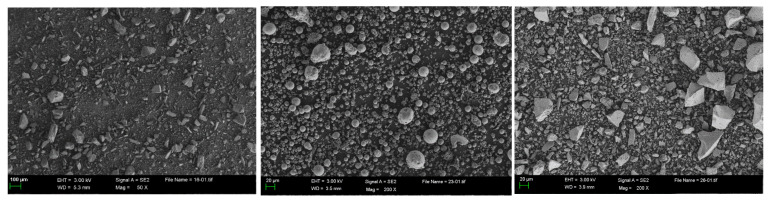
SEM pictures of ITP, FA, and LS.

**Figure 3:**
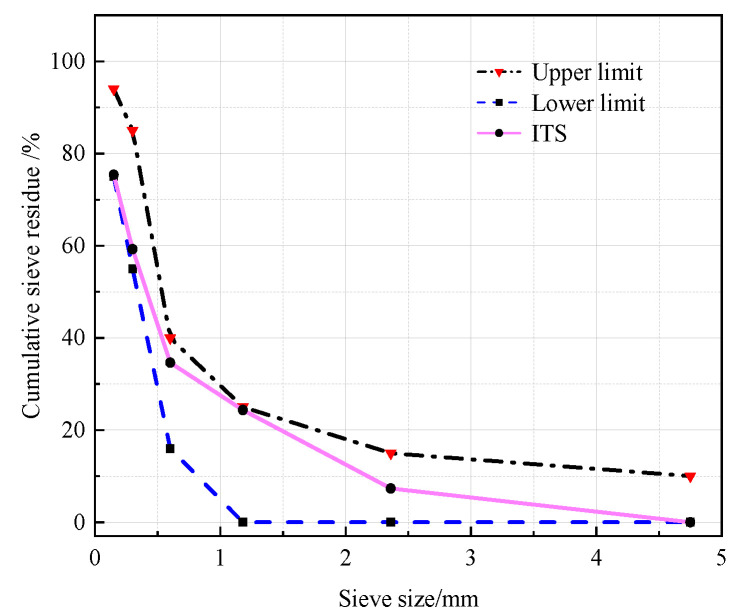
The particle gradation of ITS.

**Figure 4:**
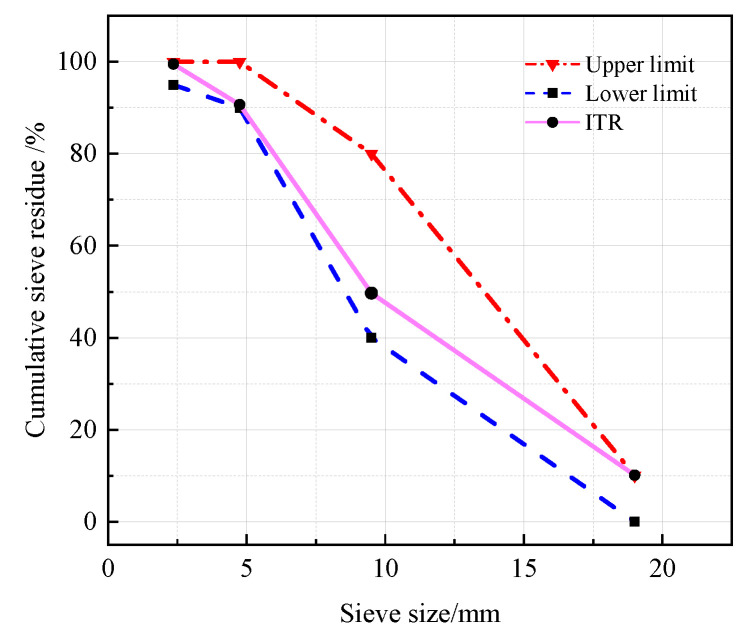
The particle gradation of ITR.

The P.O 42.5 Portland cement (OPC) was selected as the binder, meeting the Chinese standard GB 175-2007. At different CRRs, ITP, FA, and LS were used to replace OPC. The chemical compositions and specific surface areas of ITP, FA, and LS are listed in Table 1 and Table 2, respectively. The specific surface area was determined by BET method. The particle size distributions and microscopic morphology of ITP, FA, and LS are presented in Figure 1 and Figure 2, respectively. The particle size distribution was determined by Malvern Mastersizer 2000 laser particle size analyzer; the microscopic morphology was determined by ZEISS Gemini 300 scanning electron microscope. The iron tailings sand (ITS) was utilized as a fine aggregate with a fineness modulus of 2.0. The physical properties are provided in Table 3, and the particle gradation of ITS is shown in Figure 3. The iron tailings rock (ITR), with a particle size scope of 5 to 20 mm, was utilized as coarse aggregate. The physical properties of ITR are provided in Table 4, and the particle gradation of ITR is reported in Figure 4. The particle gradation was determined by the Chinese standard GB/T14685-2011. The water reducer was a P-II water reducer. The performance index of water reducer is listed in Table 5. The acid was a 99.5% concentration of concentrated acetic acid. The regular tap water (drinking water) was used for mixing.

### 2.2. Mix Proportion and Preparation of Concrete

The mixed proportions of concrete are listed in Table 6. IFL-0 is the OPC without admixture; specimens IFL-1 to IFL-4 are the ternary systems composed of ITP, FA, and LS with a mixed mass ratio of 2:1:1, a fixed w/b of 0.42 and CRRs of 10%, 20%, 30%, and 40%, respectively; specimens IFL-2, IFL-5 to IFL-7 are the ternary systems composed of ITP, FA, and LS with a mixed mass ratio of 2:1:1, a fixed CRR of 20% and w/b of 0.42, 0.44, 0.46 and 0.48, respectively.

For specimen preparation, ITS and ITR were dried in the oven at 105 °C for 24 h. Before the dried ITS and ITR were placed into the blender for 1 min, the weighed OPC, ITP, FA, and LS were put into the blender for 1 min. Finally, the water and water reducer were mixed well, and all of them were dumped into the blender for 2 min. After mixing, the mixtures were put into a 100 mm × 100 mm × 100 mm mold and consolidated using a vibrating table for 30 s. The specimens were cured at a standard curing condition of 20 ± 2 °C and 95 ± 5% relative humidity for 24 h before being tested.

### 2.3. Test Methods

(1) Acid erosion: The acid erosion test consisted of 99.5% concentrated acetic acid being configured into an acidic solution with pH = 3. During the erosion process, the concentration of the saturated solution was adjusted every seven days to keep the concentration of the solution stable. The saturated solution was replaced once every 30 days, and the soaking cycle was 60 days.
(2) Compressive strength loss: The Shenzhen Universal testing equipment was used to test the concrete cube’s compressive strength in line with Chinese standard GB/T 50081-2019, and the compressive strength loss (*CSL*) was computed as follows:
  $$CSL=\frac{f_{t}-f_{S}}{f_{t}}\times 100％,$$
  where *f_t_* is the average compressive strength (MPa) of three specimens for the standard curing 28 days and *f_s_* is the average compressive strength (MPa) of three specimens for the acid erosion 60 days.
(3) Mass loss: The mass test measures the mass of the specimen after 28 days of the standard curing and the mass of the specimen after 60 days of acid erosion. The mass of the concrete was measured using an electronic scale with a precision of ±1 g. At the measurement time, the specimens were rinsed with tap water, and then dried at 50 ± 2 °C for 48 h. The mass loss (*ML*) of concrete was then determined as follows
  $$ML=\frac{W_{t}-W_{S}}{W_{t}}\times 100％,$$
  where $W_{t}$ is the average mass (g) of three specimens for the standard curing 28 days and $W_{s}$ is the average mass (g) of three specimens for the acid erosion 60 days.
(4) Apparent deterioration: The apparent deterioration test examined soaking concrete specimens in an acetic acid solution that causes apparent changes by recording the apparent changes of acid erosion on concrete specimens in the early, middle, and late stages.
(5) MIP: Taking the concrete specimen, a 15 mm thick slice of concrete was cut out with a cutter before a core was drilled and sampled using an electric drill and a hollow drill bit with an inner diameter of 8–14 mm. The sample contained no aggregate. After sampling, the specimen was immersed in anhydrous ethanol for seven days to terminate hydration. Finally, the specimen was baked for three days at a temperature of 50 ± 2 °C to obtain the sample to be tested. The pore distribution was determined using the AutoPore Iv 9510 high performance automatic mercury injection instrument.
(6) SEM: Taking the concrete specimen, a 3–5 mm thick slice of concrete was cut out with a cutter before using an electric drill to drill a core sample in the slice. After sampling, the specimen was immersed in anhydrous ethanol for seven days to terminate hydration. Then, the sample was placed in an ultrasonic cleaner to clean, and finally put into an oven at a temperature of 50 ± 2 °C for three days to obtain the sample to be tested. The micromorphology was determined by ZEISS Gemini 300 scanning electron microscope.

## 3. Results and Discussion

### 3.1. Compressive Strength

#### 3.1.1. Compressive Strength of Concrete at Different CRRs

**Figure 5:**
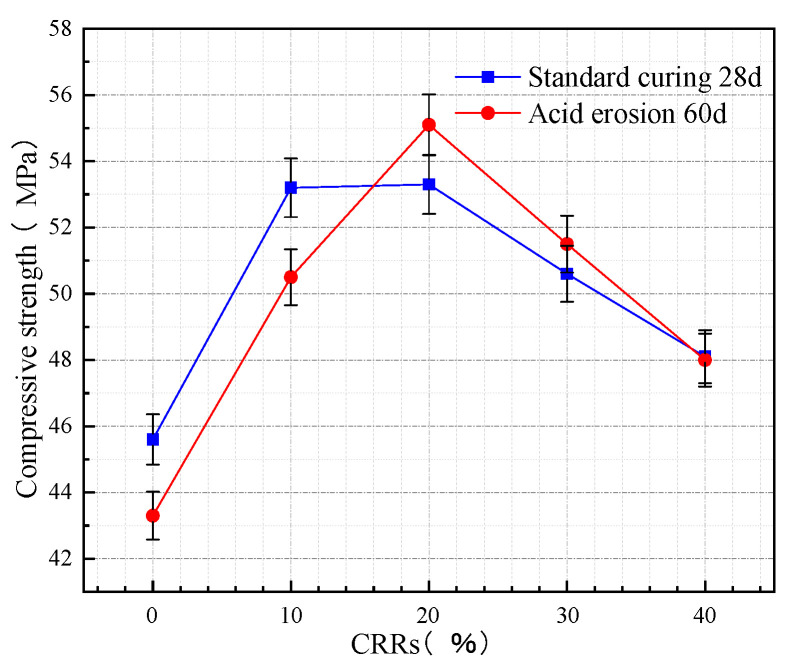
The variation of compressive strength of concrete at different CRRs.

The 28 d compressive strength of concrete after standard curing with different CRRs is shown in Figure 5. At a certain w/b, the 28 d compressive strength of concrete after standard curing first rises and subsequently drops as CRRs improve. The 28 d compressive strength of the concrete after standard curing reaches a maximum value of 53.3 MPa when the CRR is 20%, compared to IFL-2 with IFL-0, which increases by 16.9%. When the CRRs are 10%, 30%, and 40%, respectively, the 28 d compressive strength after standard curing increases by 16.7%, 11% and 5.5%, respectively, compared with IFL-0. The result indicates that the use of mineral admixtures instead of cement is beneficial to increase the compressive strength of concrete, which is consistent with the conclusion in the literature [31,32,33]. The mechanically ground ITP makes the microstructure denser, and the fine ITP exhibits the filling effect of macroaggregates [23]. Proper blending with FA and LS can improve the particle gradation, disperse in the concrete, fill the spaces inside the microstructure, and increase the compressive strength of the test block. Meanwhile, ITP, FA, and LS contain volcanically active chemical components and react with CH (Ca(OH)_2_) to form C-S-H gels [24,34], which makes the internal structure of concrete denser and enhances the compressive strength.

The acid erosion 60 d compressive strength of concrete with different CRRs is displayed in Figure 5 At a certain w/b, the acid erosion 60 d compressive strength of concrete specimens first rises and then drops with increasing CRRs. When the CRR is about 16%, the acid erosion 60 d compressive strength is the same as the standard curing 28 d compressive strength of concrete. When the CRR is below 16%, the 28 d compressive strength of concrete after standard curing is better than the acid erosion 60 d compressive strength of concrete. The highest loss of compressive strength of concrete after 60 d of acid erosion is 5.1% at a CRR of 10%. When the CRRs are higher than 16%, the acid erosion 60 d compressive strength of concrete is better than the 28 d compressive strength of concrete after standard curing. The highest rise in compressive strength of concrete in 60 days of acid erosion is 3.4% at a CRR of 20%, indicating stronger resistance to acid erosion. This is consistent with the conclusion in the literature [25] that the presence of supplementary cementitious materials lowers the detrimental effect of acids on concrete. When the CRRs are lower than 16%, the cement content is relatively large, and the acid solution mainly reacts with the hydration products generated by cement hydration, resulting in the loose expansion of the internal structure of concrete and a decrease in concrete compactness, a reduction in compressive strength. When the CRRs are higher than 16% with increasing admixture, the early activity of ITP is low [28], which reduces the hydration rate of cement and the internal pore structure of concrete. As the reaction proceeds, the volcanic ash reaction of ITP, FA, and LS with specific activity occurs [34,35], which consumes the unhydrated particles and fills the connected pores. In addition, the good volcanic ash reaction of FA and LS can improve the pore structure of concrete [24,36], enhance the amount of cementation and the cementation process, and fill capillaries and void cracks in concrete specimens. Therefore, the filling effect increases the compactness of concrete to some extent, strengthens the acid erosion resistance of concrete, and hinders the transmission of erosion media into concrete pores.

#### 3.1.2. Compressive Strength of Concrete at Different w/b

**Figure 6:**
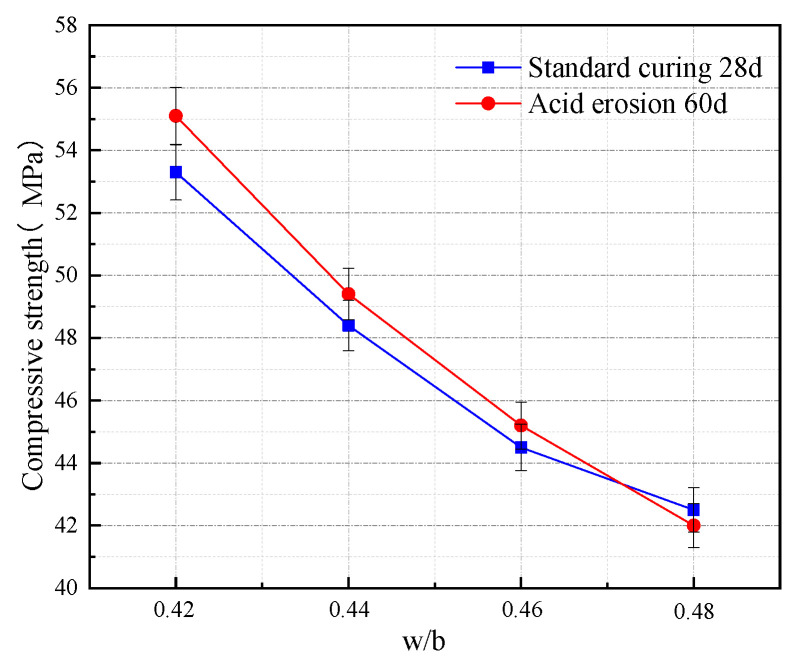
The variation of compressive strength of concrete at different w/b.

The 28 d compressive strength of concrete after standard curing with different w/b is presented in Figure 6. At a certain CRR, the 28 d compressive strength of concrete after standard curing gradually drops with the improvement of w/b. When the w/b is 0.42, the concrete specimen’s standard curing 28 d compressive strength reaches a maximum of 53.3 MPa. When the w/b increases from 0.42 to 0.44, 0.46, and 0.48, the 28 d compressive strength of concrete after standard curing decreases by 9.2%, 16.5%, and 20.3%, respectively, which is consistent with the conclusion in the literature [37] that the compressive strength of concrete drops as w/b increases. As w/b increases, the concrete’s hardening water level rises more, porosity increases, and the corresponding capillary pores increase, resulting in the concrete’s decreasing compactness. This phenomenon becomes more severe with the growth of water content, so with the rise of the w/b test block, compressive strength decreases.

The acid erosion 60 d compressive strength of concrete with different w/b is shown in Figure 6. At a certain CRR, the acid erosion 60 d compressive strength of concrete gradually decreases with the increase in the w/b. When the w/b is about 0.47, the acid erosion 60 d compressive strength is the same as the 28 d compressive strength of concrete after standard curing. When the w/b is less than 0.47, the acid erosion 60 d compressive strength of concrete is better than the 28 d compressive strength of concrete after standard curing, which shows strong resistance to acid erosion. The acid erosion 60 d compressive strength of concrete improved by 3.4% at a w/b of 0.42. When the w/b is higher than 0.47, the acid erosion 60 d compressive strength of concrete is lower than the 28 d compressive strength of concrete after standard curing, and the acid erosion 60 d compressive strength of concrete lost 1.2% at a w/b of 0.48. When the w/b is less than 0.47, the particle size of the incorporated ITP, FA, and LS is very dense, which improves the density and impermeability of the concrete and improves the filling of cement particles. The micro-aggregate effect of ITP improves the hydration environment of cement [23] and the homogeneity of the concrete. Moreover, the addition of admixtures makes up for the defect of the poor bonding surface of cement paste and aggregate [32], which hinders the erosion of concrete by acetic acid solution. As the w/b increases, the greater the degree of diffusion of the erosion medium, the faster the diffusion rate, and the erosion resistance of concrete decreases, resulting in lower compressive strength. When the w/b is too large, the water content of the concrete is high, and the water reducer releases the water in the flocculation structure generated by cement hydration. Excess water in the system occurs, thus significantly increasing the porosity of the concrete structure. An increase in the w/b also makes the internal secondary hydration incomplete. The reduction of hydration products leads to the internal system of the substantial organization becoming thin and the erosion medium more easily invaded, making the compressive strength of tangible decrease.

### 3.2. Mass Loss

#### 3.2.1. Mass Loss of Concrete at Different CRRs

**Figure 7:**
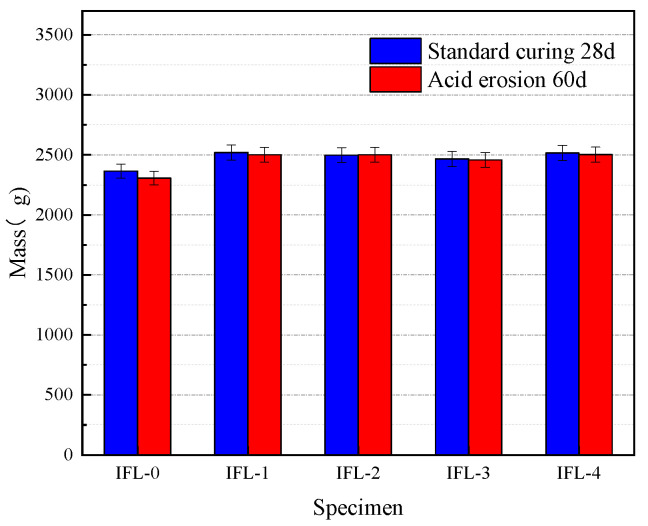
The variation of the mass of concrete at different CRRs.

The mass and ML of concrete at different CRRs is exhibited in Table 7. It can be seen that the CCRs were 0, 10%, 20%, 30%, and 40%, respectively, and the ML of concrete was 2.49%, 0.75%, −0.12%, 0.32%, and 0.56%, respectively, for a certain w/b. The concrete mass with a CRR of 20% increased by 0.12%. The result indicates that the concrete composed of ITP, FA, and LS can improve the mass of concrete after an acid erosion, which is consistent with the conclusion in the literature [25]. When the acid comes into contact with the concrete, it reacts with the hydration products and produces products that cause an increase in the mass of the concrete [38]. The mass of concrete in OPC (IFL-0) loss was 2.49%. The variation of concrete mass at different CRRs is shown in Figure 7. The mass of admixture concrete has little change, while that of OPC concrete has great change. Compared with the concrete of OPC, the overall ML of the composite admixture was smaller. From the results, the composite admixture reduced the ML of the concrete with certain acid erosion resistance.

#### 3.2.2. Mass Loss of Concrete at Different w/b

**Figure 8:**
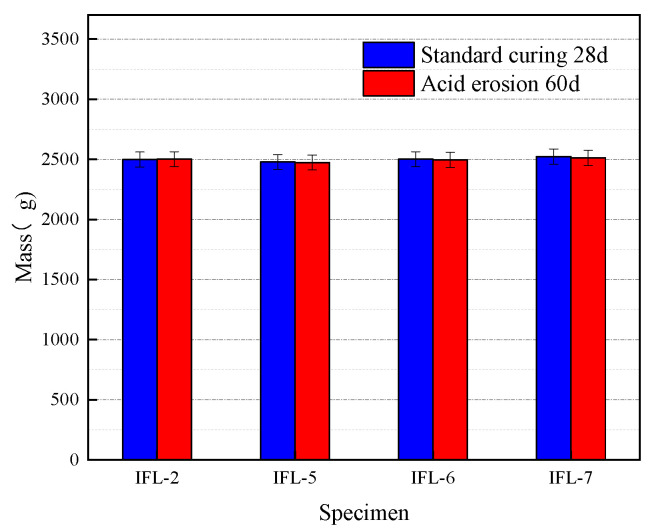
The variation of the mass of concrete at different w/b.

The mass and ML of concrete at different w/b are exhibited in Table 8. When the w/b is 0.42, 0.44, 0.46, and 0.48, respectively, the ML of concrete is −0.12%, 0.20%, 0.24%, and 0.40%, respectively. It can be seen from the results that the mass loss increases with the increase in w/b. The reason is that the higher w/b, the higher the porosity, the higher the corrosion degree and the greater ML, which is consistent with the conclusion in literature [38]. The variation of concrete mass at different w/b is shown in Figure 8. There is no change in the mass of these four specimens. Compared with the mass loss of the OPC concrete, the overall mass loss rate of the composite admixture is smaller and has certain acid erosion resistance.

### 3.3. Apparent Deterioration

**Figure 9:**
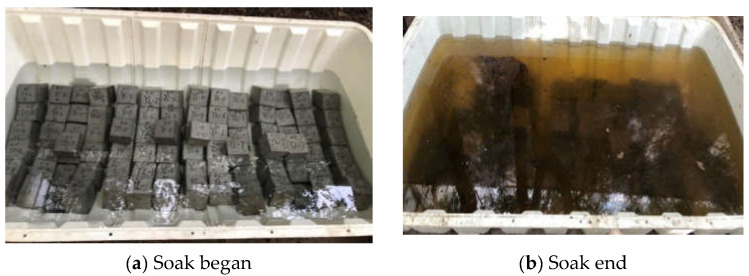
Acid-soaked concrete test block.

**Figure 10:**
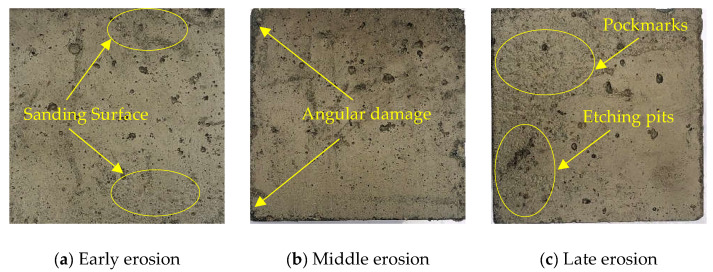
The apparent deterioration of concrete.

The process of concrete test block being soaked by acid solution is displayed in Figure 9. Deterioration of concrete surfaces occurs due to acid erosion [39]. The apparent decline of concrete is shown in Figure 10. In the early erosion, the character and corners of the concrete test block were relatively intact. Sanding only appeared in the surface layer. With time, in the middle of the erosion, the corners of the concrete test block began to be damaged, and the central part was relatively more intact. Because of the loss of the paste, the surface of the test block appeared rougher, and the overall shape became irregular. The test block surface damage is aggravated by the continuous erosion of acetic acid solution. In the late erosion, the test block surface damage deteriorates, there are different degrees of pockmarks and etching pits, and the overall pockmarks are relatively dense. The reason is that the erosion medium in acetic acid solution reacts with the hydration product and forms soluble salt, which leads to the deterioration of the surface of the concrete test block. The overall resistance of the concrete test block to the erosion of the acid solution is good, no more bottomless pits and larger cracks appear on the surface.

### 3.4. MIP

**Figure 11:**
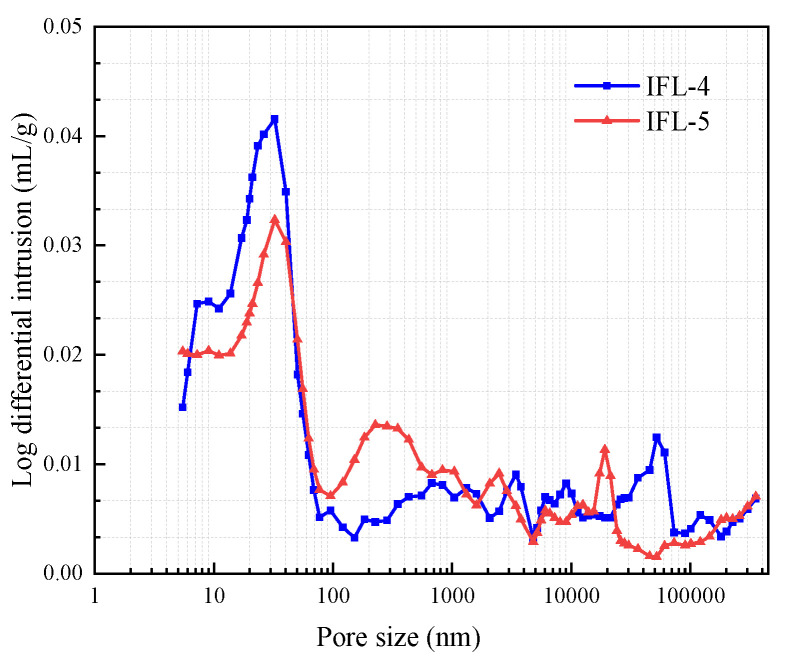
The particle pore distribution of concrete.

**Figure 12:**
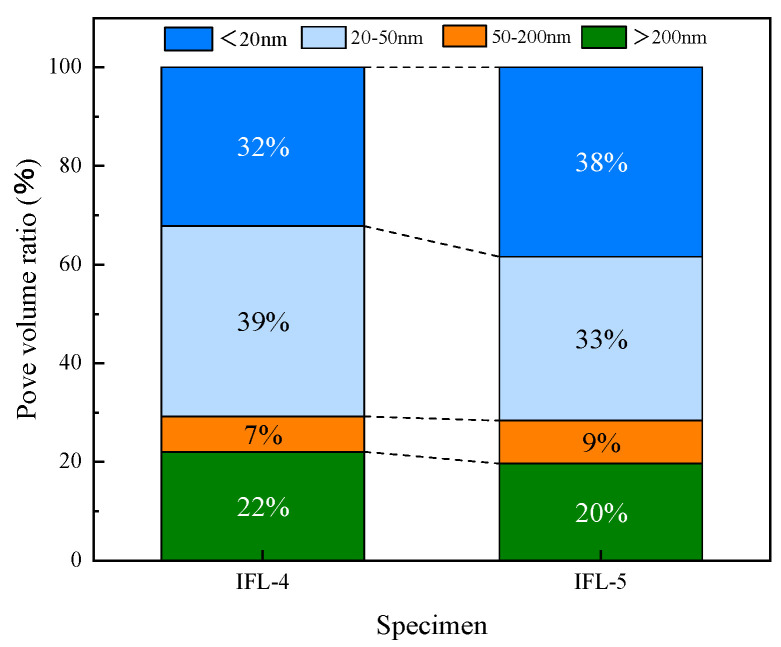
The pore volume ratio of concrete.

The particle pore distribution of concrete is displayed in Figure 11. The pore distribution was determined using the AutoPore Iv 9510 high performance automatic mercury injection instrument. The peak strength of IFL-4 is higher than that of IFL-5, implying a high peak with a large CRR. Referring to the method in literature [40], the aperture is divided into species, and the results are shown in Figure 12. IFL-4 dominant pores are 20–50 mm in diameter and account for 39% of the total; IFL-5 prevalent pores are less than 20 mm in diameter and account for 38% of the total. The increase in the number of tiny pores can improve the impermeability of concrete, indicating that IFL-5 is more resistant to acid attack than IFL-4. This finding is corroborated by the previous experimental results obtained within the context of this study, the CSL of IFL-4 is 0.2%, the CSL of IFL-5 is −2.1%, the CSL of IFL-5 is smaller; the ML of IFL-4 is 0.56%, the ML of IFL-5 is 0.2%, and the ML of IFL-5 is smaller. The variation in the pore structure of concrete is an essential indicator of mechanical properties and durability [40]. The microstructure and properties of concrete can be improved by using mineral admixtures.

### 3.5. SEM

**Figure 13:**
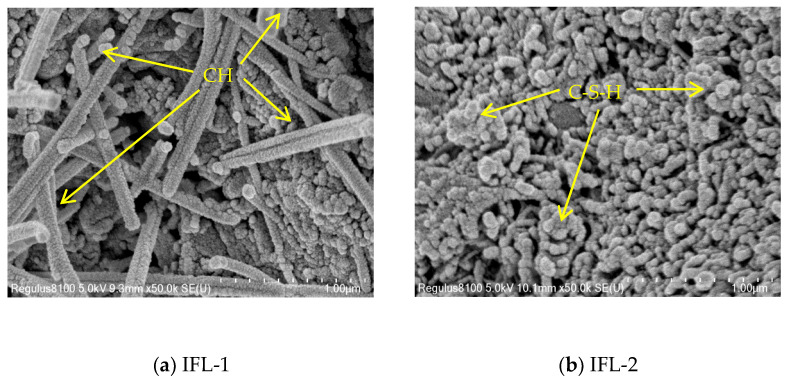

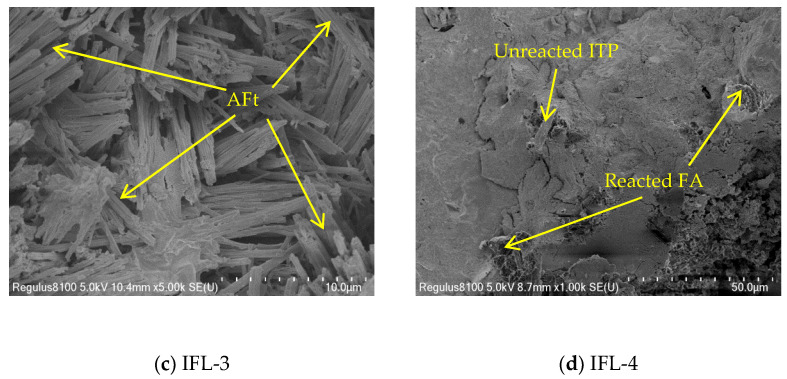
The SEM of the multi-solid waste mineral admixture concrete.

The SEM of the multi-solid waste mineral admixture concrete is presented in Figure 13. In the hardened slurry, it can be observed that a large number of rod-like CH are shown in Figure 13a, clustered C-S-H gels are shown in Figure 13b, the needle-like AFt are presented in Figure 13c. Figure 13d shows reacted FA and unreacted ITP. A large amount of C-S-H gels with Aft crystals makes the concrete internally dense [41], less porous, with uniform pore distribution, and more viscous, which is beneficial to enhance the compressive strength and permeability of concrete. The unreacted ITP and reacted FA are depicted in Figure 13d. This indicates that the hydration ability of FA is a little stronger than that of ITP. The primary role of FA is to enhance the performance of concrete by facilitating secondary hydration reactions. In contrast, the primary function of ITP is the filling effect, which corresponds to the experimental results that the previous admixture can enhance the compressive strength of concrete. Using ITP, FA, and LS as mineral admixtures to replace some OPC, concrete with excellent properties can be made.

## 4. Conclusions

The study focuses on the durability of ITP-FA-LS ternary mineral admixture concrete under acetic acid erosion. The compressive strength, compressive strength loss, and mass loss of concrete were analyzed by different CRRs and different w/b, and the microstructure of concrete was conducted using MIP and SEM. The following conclusions can be derived:

(1) When the CRR is 20%, the standard curing 28 d compressive strength of concrete is the highest, which is higher than OPC concrete. When the w/b is 0.42, the standard curing 28 d compressive strength of concrete is the highest. The activity of ITP is significantly increased after grinding. Appropriate mixing with FA and LS can help with the particle gradation and produce a micro-aggregate effect. A volcanic ash reaction occurs, which has a strong hydration reaction ability and makes the compressive strength of concrete increase.
(2) Concrete eroded with acetic acid solution after 60 days. When the CRR is greater than 16%, especially at 20%, the concrete strength increase rate after acid erosion 60 d was the largest and showed strong acid erosion resistance. When the w/b is less than 0.47, especially at 0.42, the concrete strength increase rate after acid erosion 60 d is the largest and shows strong acid erosion resistance. The good volcanic ash activity of FA and LS improved the pore structure of concrete, and improved the amount of cementation and cementation process. The filling effect increased the compactness of concrete to a certain extent, strengthened the acid penetration resistance of concrete, and hindered the transport of ions in acetic acid solution in the pores of the concrete.
(3) Acidic solution erosion deteriorates the concrete surface. At the same time, there is a loss of mass of concrete, which is minimal when the CRRs is 20% and minimal when the w/b is 0.42; all of them are smaller than the ML of the OPC concrete specimen. The ternary mineral admixture system consisting of ITP, FA, and LS resulted in more delicate pores and a denser structure of concrete, which effectively hindered the erosion of concrete by acetic acid solution.
(4) The mineral doping system reduces the permeability of the erosion medium. To a certain extent, it can alleviate the erosion of acid solution concrete through MIP and SEM, which confirms that the concrete has lower internal porosity and better density. The ITP-FA-LS ternary mineral admixture concrete has better acid erosion resistance than OPC concrete.

## Figures and Tables

**Table 1:** Chemical compositions of ITP, FA, and LS.

Materials	Chemical Composition (%)									
	SiO_2_	Al_2_O_3_	CaO	Fe_2_O_3_	MgO	SO_3_	K_2_O	Na_2_O	TiO_2_	MnO
ITP	62.26	4.78	7.77	14.37	6.33	0.48	1.39	1.34	0.53	0.21
FA	60.06	25.09	2.93	6.74	0.86	0.26	1.61	0.11	1.49	0.16
LS	54.55	25.38	6.44	1.41	0.60	6.05	0.70	0.10	0.03	0.07

**Table 2:** Specific surface area of ITP, FA, and LS.

Materials	ITP	FA	LS
Specific surface area (m^2^/kg)	1290	1391	13,627

**Table 3:** The physical properties of ITS.

Stone Powder Content (%)	Sludge Content (%)	Stacking Density (kg/m^3^)	Apparent Density (kg/m^3^)	Mass Loss (%)	Crushing Index (%)
4.9	0	1620	2560	4	22

**Table 4:** The physical properties of ITR.

Mud Content (%)	Sludge Content (%)	Needle and Flake Content (%)	Crushing Index (%)	Stacking Density (kg/m^3^)	Apparent Density (kg/m^3^)
0.1	0	3	7	1610	2630

**Table 5:** The performance index of WR.

Water Reduction Rate (%)	Water Secretion Rate (%)	Gas Content (%)	28 d Shrinkage Ratio (%)	Solids Content (%)
27	24	3.9	103	12.04

**Table 6:** The mix proportions of concrete.

Specimen	Dosage of Each Component (kg/m^3^)							
	Water	OPC	ITP	FA	LS	ITS	ITR	Water Reducer
IFL-0	176.4	420	0	0	0	740	1 110	5.2
IFL-1	176.4	378	21	10.5	10.5	740	1 110	5.2
IFL-2	176.4	336	42	21	21	740	1 110	5.2
IFL-3	176.4	294	63	31.5	31.5	740	1 110	5.2
IFL-4	176.4	252	84	42	42	740	1 110	5.2
IFL-5	184.8	336	42	21	21	740	1 110	5.2
IFL-6	193.2	336	42	21	21	740	1 110	5.2
IFL-7	201.6	336	42	21	21	740	1 110	5.2

**Table 7:** The mass and ML of concrete at different CRRs.

Specimen	CRRs (%)	w/b	Standard Curing 28 d (g)	Acid Erosion 60 d (g)	ML (%)
IFL-0	0	0.42	2365	2306	2.49
IFL-1	10	0.42	2520	2501	0.75
IFL-2	20	0.42	2498	2501	−0.12
IFL-3	30	0.42	2466	2458	0.32
IFL-4	40	0.42	2516	2502	0.56

**Table 8:** The mass and ML of concrete at different w/b.

Specimen	CRRs (%)	w/b	Standard Curing 28 d (g)	Acid Erosion 60 d (g)	ML (%)
IFL-2	20	0.42	2498	2501	−0.12
IFL-5	20	0.44	2478	2473	0.20
IFL-6	20	0.46	2501	2495	0.24
IFL-7	20	0.48	2523	2513	0.40

## Data Availability

All data is provided in the manuscript.
